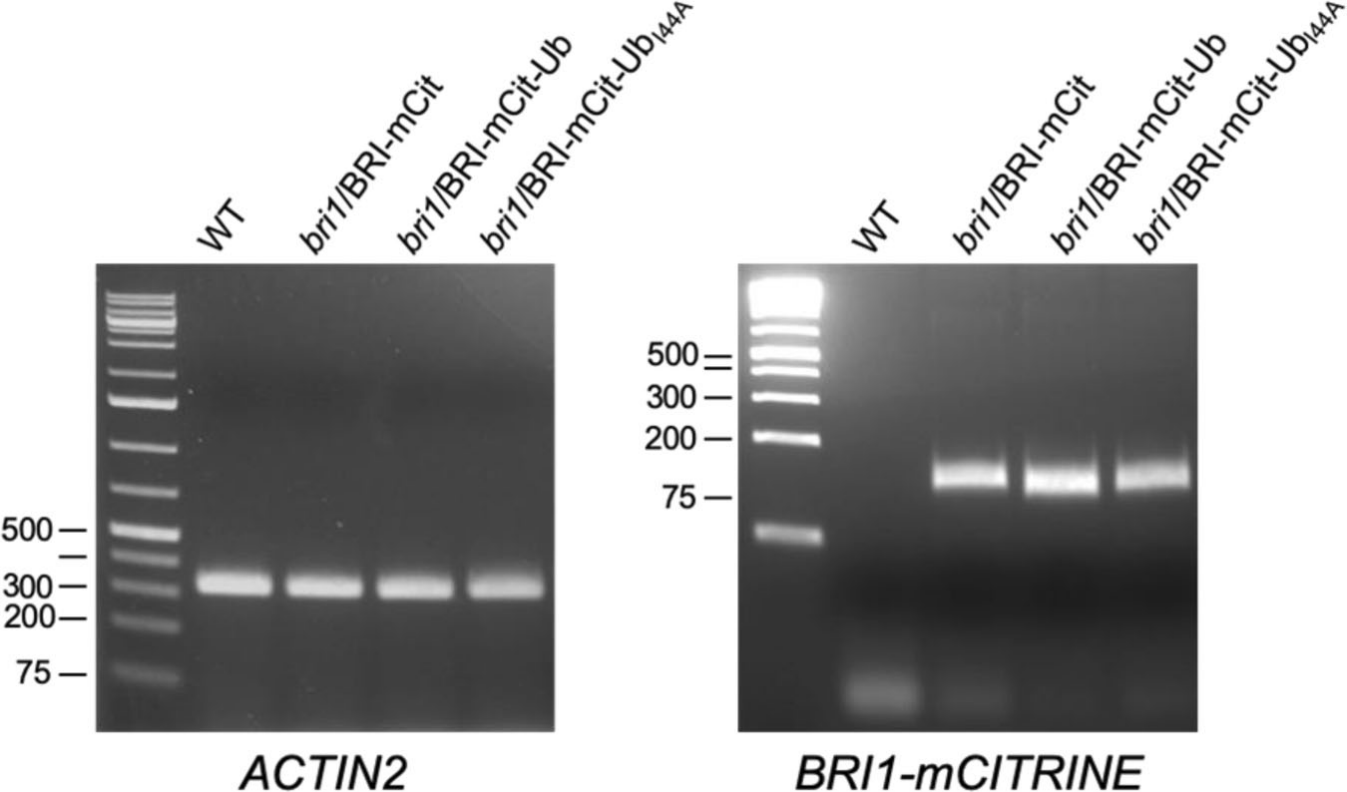# Author Correction: Internalization and vacuolar targeting of the brassinosteroid hormone receptor BRI1 are regulated by ubiquitination

**DOI:** 10.1038/s41467-021-23193-x

**Published:** 2021-05-17

**Authors:** Sara Martins, Esther M. N. Dohmann, Anne Cayrel, Alexander Johnson, Wolfgang Fischer, Florence Pojer, Béatrice Satiat-Jeunemaître, Yvon Jaillais, Joanne Chory, Niko Geldner, Grégory Vert

**Affiliations:** 1 Institute for Integrative Biology of the Cell (I2BC), CNRS, CEA, Paris-Sud University, Avenue de la Terrasse, 91190 Gif-sur-Yvette, France; 2 Institut des Sciences du Végétal, Unité Propre de Recherche 2355, Centre National de la Recherche Scientifique, Saclay Plant Sciences, Avenue de la Terrasse, 91190 Gif-sur-Yvette, France; 3 Department of Plant Molecular Biology, University of Lausanne, UNIL-Sorge, 1015 Lausanne, Switzerland; 4 The Salk Institute for Biological Studies, 10010 North Torrey Pines Road, La Jolla, California, 92037 USA; 5 Protein Crystallography Core Facility, Ecole Polytechnique Fédérale de Lausanne, SV 3827 Station 19, 1015 Lausanne, Switzerland; 6 Laboratoire de Reproduction et Développement des Plantes, INRA, CNRS, ENS Lyon, Université de Lyon, 46 allée d’Italie, 69364, Lyon 07, France; 7 Howard Hughes Medical Institute, The Salk Institute for Biological Studies, 10010 North Torrey Pines Road, La Jolla, California, 92037 USA

**Keywords:** High-harmonic generation, Atomic and molecular interactions with photons, Attosecond science

Correction to: *Nature Communications* 10.1038/ncomms7151, published online 21 January 2015.

This article contains an error in Supplementary Fig. 2b. The gel image in the lower panel labelled as *ACT2* was duplicated from a previous gel image labelled as *Ubq10* in Figure S5 of a prior publication^1^. The RT-PCR experiment in question has since been repeated to analyze *ACTIN2* and *BRI1m-CITRINE* expression in the WT, *bri1*/BRI1-mCitrine, *bri1*/BRI1-mCitrine-Ub and *bri1*/BRI1-mCitrine-Ub_I44A_ genotypes described in the original article. 27 cycles of PCR amplification was performed using primers 5′-GCCCAGAAGTCTTGTTCCAG-3′ and 5′-TCATACTCGGCCTTGGAGAT-3′ for *ACTIN2* and 5′-GACTTCTTCAAGTCCGCCATG-3′ and 5′-GTCCTCCTTGAAGTCGATGC-3′ for *mCITRINE*. cDNA was prepared as described in the original article, and PCR products were ran on 2% and 4% agarose gels for *ACTIN2* and *BRI1-mCitrine*, respectively. The result of the new experiment appears below as Fig. 1.